# Formononetin inhibits tumor growth by suppression of EGFR-Akt-Mcl-1 axis in non-small cell lung cancer

**DOI:** 10.1186/s13046-020-01566-2

**Published:** 2020-04-10

**Authors:** Xinyou Yu, Feng Gao, Wei Li, Li Zhou, Wenbin Liu, Ming Li

**Affiliations:** 1 Cell Transplantation and Gene Therapy Institute, The Third Xiangya Hospital, Central South University, Changsha, Hunan 410013 P.R. China; 2 Shandong Lvdu Bio-Industry Co Ltd, Binzhou, Shandong 256600 P.R. China; 3 Department of Ultrasonography, The Third Xiangya Hospital of Central South University, Changsha, Hunan 410013 P.R. China; 4 Department of Radiology, The Third Xiangya Hospital of Central South University, Changsha, Hunan 410013 P.R. China; 5 Department of Pathology, Xiangya Hospital of Central South University, Changsha, Hunan 410008 P.R. China; 6 Department of Pathology, Hunan Cancer Hospital, Changsha, Hunan 410013 P.R. China; 7 Changsha Stomatological Hospital, Changsha, Hunan 410004 P.R. China; 8 School of Stomatology, Hunan University of Chinese Medicine, Changsha, Hunan 410208 P.R. China

**Keywords:** Non-small cell lung cancer, Formononetin, Epidermal growth factor receptor, Mcl-1, Ubiquitination

## Abstract

**Background:**

Epidermal growth factor receptor (EGFR) activating mutations play crucial roles in the tumorigenesis of human non-small cell lung cancer (NSCLC). The mechanism regarding how EGFR signaling regulates myeloid cell leukemia sequence 1 (Mcl-1) protein stability and ubiquitination remains undefined.

**Methods:**

MTS assay was used for natural product library screening. The effect of formononetin (Formo) on NSCLC cells was determined by MTS assay and soft agar assay. Molecular modeling was performed to analyze the potential different binding modes between Formo and EGFR WT or mutants. Mcl-1 protein level and the inhibitory effect of Formo on EGFR signaling were examined by immunoblot, in vitro kinase assay, in vitro pulldown and ATP competition assays, co-immunoprecipitation assay, ubiquitination analysis, in vivo xenograft model, and immunohistochemical staining.

**Results:**

Formo was identified as an EGFR inhibitor by a 98 commercially available natural product screening. Formo suppresses WT and mutant EGFR kinases activity in vitro*,* ex vivo, and in vivo. Molecular modeling indicates that Formo docks into the ATP-binding pocket of both WT and mutant EGFR. Formo inhibits EGFR-Akt signaling, which in turn activates GSK3β and promotes Mcl-1 phosphorylation in NSCLC cells. Treatment with Formo enhances the interaction between Mcl-1 and SCF^Fbw7^, which eventually promotes Mcl-1 ubiquitination and degradation. Depletion of either GSK3β or SCF^Fbw7^ compromised Formo-induced Mcl-1 downregulation. Finally, Formo inhibits the in vivo tumor growth in a xenograft mouse model.

**Conclusion:**

This study highlights the importance of promoting ubiquitination-dependent Mcl-1 turnover might be an alternative strategy to enhance the anti-tumor efficacy of EGFR-TKI.

## Background

Non-small cell lung cancer (NSCLC) is one of the most lethal cancers. Epidermal growth factor receptor (EGFR) activating mutations are considered as a driving force for tumorigenesis of some NSCLC. Over 90% of EGFR activating mutations which occur in both Asian and Western NSCLC patient present as an exon 19 deletion (60%) or exon 21 point mutation (30%) [1–3]. Targeting therapy with the tyrosine kinase inhibitors (TKIs), such as gefitinib and erlotinib, has become the first-line treatment for these patients with EGFR activating mutations. However, most patients who initially respond to TKIs eventually develop acquired resistance. Beyond c-Met amplification, previous studies reveal that over 60% of acquired resistant cases associated with the emergence of a secondary mutation of EGFR, T790M. The threonine to methionine mutation, which occurs in the EGFR tyrosine kinase domain, promotes ATP binding affinity and attenuates the interaction between EGFR tyrosine kinase domain and the first-generation reversible EGFR-TKIs [4, 5]. Osimertinib represents the third-generation EGFR-TKIs, which irreversibly inhibit EGFR activating mutations, overcomes EGFR T790M secondary mutation conferred acquired resistance to first- and second-generation TKIs. Although osimertinib significantly improved the progression-free survival (PFS) of NSCLC patients with EGFR T790M mutation, the development of acquired resistance to the third-generation EGFR-TKIs has already been described and increased in the clinic [6–8]. However, the precise mechanisms mediating resistance to osimertinib remain largely unknown, and the strategies to overcome osimertinib resistance are still limited.

Myeloid cell leukemia sequence 1 (Mcl-1) is a member of the pro-survival Bcl-2 family that negatively regulates the mitochondrial apoptotic pathway. Overexpression or amplification of Mcl-1 is frequently observed in human cancers and associated with poor prognosis. Inhibition of Mcl-1 sensitizes chemo/radiotherapy induced apoptosis in multiple cancer models [9–11]. Recent studies showed that Mcl-1 is upregulated by EGFR signaling. For example, EGF stimulation enhances Mcl-1 transcription in a transcription factor Elk-1 dependent manner [12]. In EGFR mutant NSCLC cells, hyperactivation of mTORC1 increased Mcl-1 mRNA level and conferred EGFR TKI resistance [13]. The mechanisms regarding EGFR activation and Mcl-1 transcription were well studied previously. However, the mechanisms underlying how EGFR signaling regulates Mcl-1 protein stability, as well as ubiquitination, remains elusive.

Previous studies have demonstrated that the natural compound, formononetin (C_16_H_12_O_4_), exhibits significant anti-tumor potentials against human cancers [14, 15]. The evidence from in vitro and in vivo studies reveal that Formo acts as a novel anti-tumorigenic agent to induce cell cycle arrest, apoptosis, anti-angiogenesis, and metastasis in a panel of solid tumors, including lung cancer [16], colorectal cancer [17], breast cancer [18], and gastric cancer [19]. The mechanism studies indicate that deactivation of protein kinases and signal transduction, or dysfunction of oncogenetic-related transcription factors, are involved in Formo-induced anti-tumor activities [14, 15]. However, the inhibitory efficacy of Formo on EGFR signaling, and the anti-tumor effect of Formo on both osimertinib sensitive and resistant NSCLC cells, is not clear.

In the present study, by the screening of a natural products library, we identified Formo, a flavonoid derivative [18], as a potential anti-tumor agent for use in NSCLC therapy. We investigated the therapeutic effect using NSCLC cell lines and determined the underlying mechanism of action.

## Materials and methods

### Reagents and antibodies

The screened compound library (L1400) was a product of Selleck Chemicals (Houston, TX). The inhibitors, including Necrostatin-1, GSK’872, z-VAD-fmk, MG132, SB216763, PD98059, and LY294002, were purchased from Selleck Chemicals. Cell culture medium, Fetal Bovine Serum (FBS), and supplements were purchased from Invitrogen (Grand Island, NY). Primary antibodies against EGFR (#4267, 1:1000), ERK1/2 (#9102, 1:1000), Akt (#4691, 1:1000), p-EGFR-Tyr1068 (#3777, 1:1000), p-ERK1/2-Thr202/Tyr204 (#4370, 1:1000), p-Akt-Ser473 (#4060, 1:1000), PARP (#9532, 1:1000), cleaved-caspase 3 (#9664, 1:1000), Bax (#14796, 1:1000), VDAC1 (#4661, 1:2000), cytochrome C (#11940, 1:1000), Mcl-1 (#39224, 1:1000), ubiquitin (#3936, 1:1000), ubiquitin (#43124, 1:1000), α-Tubulin (#2125, 1:5000), GSK3β (#12456, 1:1000), and β-actin (#3700, 1:10000) were purchased from Cell Signaling Technology, Inc. (Danvers, MA). Flag-tag (F3165, 1:5000) antibody was obtained from Sigma Aldrich (St. Louis, MO). Antibodies against FBW7 (ab109617, 1:1000) and FBW7 (ab187815, 1:1000) were obtained from Abcam (Cambridge, UK). Antibodies for immunohistochemistry staining (IHC), including anti-ki67 (ab15580, 1:300) and anti-p-Akt (ab81283, 1:100) were obtained from Abcam. Anti-p-EGFR (#3777, 1:200), and anti-Mcl-1 (#39224, 1:100) were purchased from Cell Signaling Technology, Inc.

### Cell culture

Human NSCLC cells, including HCC827 (EGFR Del E746-A750), H3255 (EGFR L858R), H1975 (EGFR L858R/T790M), A549 (EGFR WT), and H1299 (EGFR WT) were purchased from American Type Culture Collection (ATCC, Manassas, VA). Cells were maintained at 37 °C in a humidified incubator with 5% CO_2_ according to ATCC protocols. The osimertinib acquired resistance cell line HCC827 OR was established in our laboratory by exposing HCC827 cells to gradually increasing concentration of osimertinib for approximately 5 months (starting at 5 nM and ending with 500 nM). All NSCLC cells were subjected to mycoplasma analysis and cytogenetically tested before being frozen. The immortalized epithelial cells NL20 and HBE were purchased from ATCC and cultured following the standard protocols. Ba/F3 cell was obtained from Cell Engineering Division/RIKEN BioResource Center (Tsukuba, Ibaraki, Japan) and maintained in RPMI1640 + 10% FBS + 10% WEHI-3 cell conditioned medium according to instructions provided. Lipofectamine® 2000 (Thermo Fisher Scientific) transfection reagent was used for plasmid transfection following the manufacturer’s instructions.

### Immunoblotting

Immunoblotting (IB) was performed as described previously [20]. Whole-cell lysate was prepared with RIPA buffer (10 mM Tris-Cl (pH 8.0), 1 mM EDTA, 0.5 mM EGTA, 1% Triton X-100, 0.1% sodium deoxycholate, 0.1% SDS. 140 mM NaCl) supplied with phosphatase and protease inhibitors, BCA kit (#23225, Thermo Fisher Scientific) was used for protein concentration. The whole-cell lysate was boiled with loading buffer and subjected to SDS-PAGE gel electrophoresis and antibody hybridization. The target proteins were visualized by chemiluminescence (Amersham Biosciences, Piscataway, NJ).

### Isolation of subcellular fractions

The Mitochondria Isolation Kit (#89874, Thermo Fisher Scientific) was used for subcellular fractions preparation according to the standard instructions.

### MTS assay

NSCLC cells were seeded (2.5 × 10^3^ /well/100 μL) in 96-well plates and treated with Formo or osimertinib, as indicated. Cell viability was examined using MTS assay (Promega, Madison, WI) following the instructions provided.

### Anchorage-independent cell growth

The anchorage-independent cell growth assay was performed as described previously [21]. Briefly, the Eagle’s basal medium containing 0.6% agar, 10% FBS, and different concentration of Formo or osimertinib was loading to a six-well plate as an agar base. Cells were then counted at the concentration of 8000 cells/ml with the Eagle’s basal medium containing 10% FBS, 0.3% agar, Formo, or osimertinib, followed by overlaid into the six-well plate containing a 0.6% agar base. The cultures were maintained in a 37 °C, 5% CO_2_ incubator for 2 weeks and counted using a microscope.

### Stable lines generation

EGFR cDNA clones, including WT EGFR, L858R EGFR, L858R/T790M EGFR, and Del E746-A750 EGFR, were subcloned into the lentivirus vector by SgfI and MluI. The Lentiviral Packaging Kit (TR30037, Origene) was used for virus package in 293 T cells. The Ba/F3 cells were infected with lentivirus together with 8 μg/mL polybrene for 24 h. Two days later, 2 μg/mL puromycin was added to the cell culture medium and maintained for another 7 days for stable cell selection.

### In vitro EGFR kinase assay

The recombinant active WT EGFR, L858R EGFR, L858R/T790M EGFR, and Del E746-A750 EGFR were purchased from Millipore. The in vitro EGFR kinase assay was performed as described previously [22]. Briefly, active EGFR (100 ng) was mixed with various doses of Formo or 100 nM osimertinib. The reaction was incubated with 500 μM angiotensin II for 5 min at room temperature, followed by incubation with 10 μL of ATP mixture (25 mM MgAc and 0.25 μM ATP containing 10 μCi [γ-32P] ATP) for 15 min at 30 °C and then 25 μL of the reaction mixture was transferred onto P81 papers. The papers were washed with 0.75% phosphoric acid twice and then with acetone once. The radioactive incorporation was determined using a scintillation counter.

### In vitro pulldown and ATP competition assays

The in vitro pulldown and ATP competition assays were performed as described previously [23]. Formo-Sepharose 4B beads were prepared following the protocol provided by GE Healthcare Biosciences. NSCLC cell lysate (500 μg) or an active kinase with different concentrations of ATP was incubated with Formo-Sepharose 4B beads or Sepharose 4B beads only in reaction buffer (50 mM Tris-HCl (pH 7.5), 150 mM NaCl, 5 mM EDTA, 1 mM DTT, 0.01% Nonidet P-40, 0.02 mM phenylmethylsulfonyl fluoride, 1 × protease inhibitor mixture and 2 μg/mL bovine serum albumin) at 4 °C with gentle rocking overnight, followed by washing with washing buffer (50 mM Tris-HCl (pH 7.5), 150 mM NaCl, 5 mM EDTA, 1 mM DTT, and 0.01% Nonidet P-40, and 0.02 mM phenylmethylsulfonyl fluoride) 5 times. Protein binding was analyzed by IB.

### Molecular Modeling

#### Homology modeling

The three-dimensional structure of EGFR with exon 19 deletion mutant (residues E746–A750) was modeled based on the wild type (WT) crystal structure of EGFR using Modeller [24]. Through extensive analysis of the deposited structures in Protein Data Bank (PDB) [25], the crystal structure of EGFR (PDB: 4JR3) was used as the template for homology modeling. Ten models were generated and evaluated with the Discrete Optimized Protein Energy (DOPE) score implemented in Modeller. Finally, the best model was adapted for the subsequent docking studies.

#### Molecular docking

The structures of WT EGFR (PDB: 4JR3), L858R EGFR (PDB: 2ITV), L858R/T790M EGFR (PDB: 3W2P), and exon 19 deletion EGFR were prepared, including filling in missing side chains, adding hydrogens and minimizing heavy atoms with default parameters using Protein Preparation Wizard in Schrödinger Suite 2013. Then the structure file of the ligand, Formo, was well pretreated in LigPrep, and docking was performed based on the standard precision mode of Glide with default settings. Docking poses for each receptor-ligand complex were then analyzed for binding modes, and final figures were generated using PyMOL. Hydrogen bond lengths were defined as the distance between the donor and acceptor atom centers.

#### Refine and MM-GBSA

Prime was employed to further refine the binding pose and calculate binding free energy (i.e. ΔG) by the MM-GBSA method using the Prime MM-GBSA module in Schrödinger Suite 2013. Residues with distances from the ligand within 12.0 Å were set as flexible. Other settings were kept in default.

### Ubiquitination assay

The ubiquitination assay was performed as described previously [26]. Cells were lysed with modified RIPA buffer containing 1% SDS (20 mM NAP, pH 7.4, 150 mM NaCl, 1% Triton, and 0.5% Sodium-deoxycholate), protease inhibitors, and 10 mM N-Ethylmaleimide (NEM). The lysates were sonicated for 30 s and boiled at 95 °C for 15 min, then diluted with 0.1% SDS containing RIPA buffer and centrifuged at 16000×g for 15 min. The supernatant was transferred to a new tube and incubated with Mcl-1 antibody plus protein A-Sepharose beads overnight at 4 °C. Beads were washed and subjected to IB analysis.

### In vivo tumor growth

All mice experiments were performed according to strict guidelines established by the Medical Research Animal Ethics Committee, Central South University, China. NSCLC cells, including HCC827 cells (2 × 10^6^), H1975 (1 × 10^6^), A549 (2 × 10^6^) and H3255 (2 × 10^6^) were counted and suspended in 100 μL RPMI-1640 medium and inoculated s.c. into the right flank of 6-week-old female athymic nude mice (6 mice per group). Formo (10 mg/kg) or vehicle control was administrated daily by i.p. Injection when the tumor volume reached 100 mm^3^, whereas osimertinib (2 mg/kg) was initiated and repeated daily by oral gavage. Mouse body weight was recorded, and tumor volume was determined by caliper. Tumor volume was calculated following the formula of A × B2 × 0.5, wherein A is the longest diameter of the tumor, B is the shortest diameter, and B2 is B squared.

### Immunohistochemical (IHC) staining

IHC staining was performed as described previously [27]. Briefly, tissue sections from xenograft tumor tissues were deparaffinized and rehydrated. The slide was unmasked by submersion into boiling sodium citrate buffer (10 mM, pH 6.0) for 10 min, followed by treating with 3% H_2_O_2_ for 10 min at room temperature. The tissue slide was blocked with 50% goat serum albumin in a humidified chamber at room temperature for 1 h, then incubated with the primary antibody at 4 °C overnight. After hybridized with the second antibody for 45 min at room temperature, the slide was incubated with DAB substrate for target protein visualization. Hematoxylin was used for counterstaining.

### Blood analysis

Mouse whole blood (200 μL) was collected in EDTA-coated tubes via cardiac puncture. The count for red blood cells (RBC) and white blood cells (WBC), hemoglobin (Hb), alanine aminotransferase (ALT), aspartate aminotransferase (AST), and blood urea nitrogen (BUN) were analyzed at the Laboratory of the Third Xiangya Hospital (Changsha, China).

### Statistical analysis

Statistical analyses were performed using SPSS (version 16.0 for Windows, SPSS Inc., Chicago, IL, USA) and GraphPad Prism 5 (GraphPad 5.0, San Diego, CA, USA). The quantitative data were expressed as means ± SD. Significant differences were determined by the Student t-test or ANOVA. A probability value of less than 0.05 was used as the criterion for statistical significance.

## Results

### Formononetin (Formo) inhibits both osimertinib sensitive and resistant NSCLC cells

**Fig. 1:**
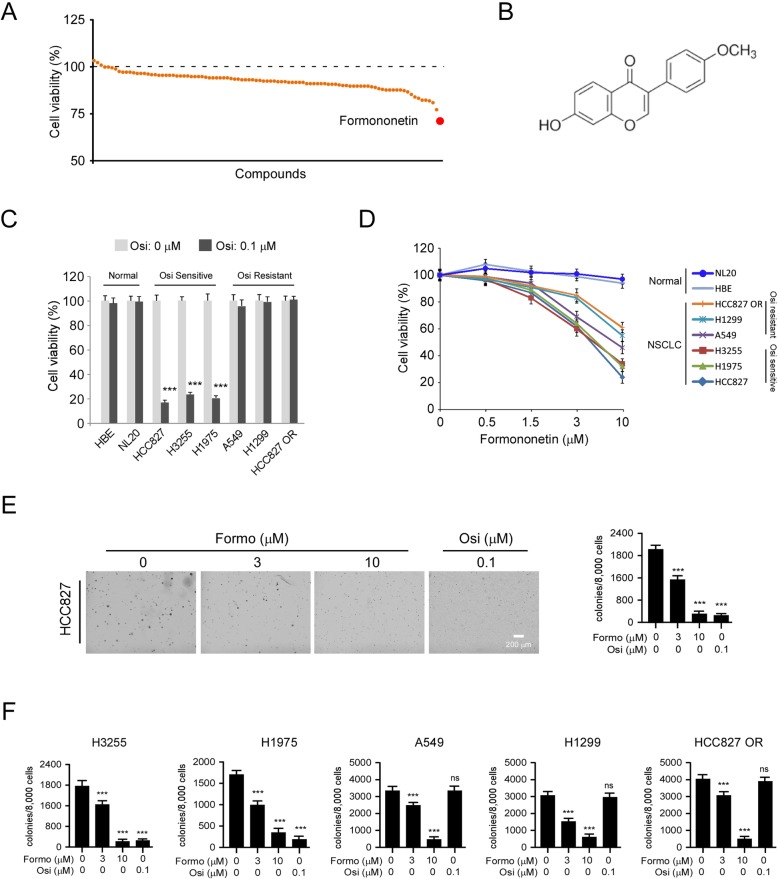
Formononetin (Formo) is a candidate natural product that suppresses non-small cell lung cancer (NSCLC) cells. **a**, The effect of 98 screened compounds on the cell viability of HCC827 cells. HCC827 cells were treated with 3 μM of commercially available natural product library for 24 h. Cell viability was examined by MTS assay. **b**, The chemical structure of Formo. **c**, The effect of osimertinib (Osi) on immortalized epithelial cells NL20 and HBE, and a panel of NSCLC cells, cell viability was determined by MTS assay. **d**, MTS assay analysis of the cell viability of NL20, HBE, and a panel of NSCLC cells with different doses of Formo treatment. **e**, The effect of Formo on anchorage-independent cell growth of HCC827 cells. HCC827 cells were treated with Formo or Osi, anchorage-independent cell growth was analyzed by soft agar assay. Left, the representative images of colony formation. Right, qualification analysis of the colony number. **f**, The effect of Formo on anchorage-independent cell growth of NSCLC cells. H3255, H1975, A549, H1299, and HCC827 OR cells were treated with Formo or Osi, anchorage-independent cell growth was analyzed by soft agar assay. ***p < 0.001. ns, not statistically significant. Scale bar, 200 μm

To discover natural products that can inhibit NSCLC cells, we screened a library of 98 commercially available compounds (Supplementary Table 1) using MTS assay. Our data indicated that only Formo decreased the cell viability of HCC827 cells over 20% at the concentration of 3 μM (Fig. 1a and b). To confirm the anti-tumor effect, we examined the inhibitory effect of Formo on osimertinib sensitive cells, HCC827 (EGFR Del E746-A750), H3255 (EGFR L858R), and H1975 (EGFR L858R/T790M), and osimertinib resistant cells A549 (EGFR WT), H1299 (EGFR WT), and HCC827 OR (osimertinib acquired resistant). The MTS result showed that osimertinib only decreased the cell viability of osimertinib sensitive NSCLC cells, but not the resistant NSCLC cells or the immortalized lung epithelial cells HBE and NL20 (Fig. 1c). In contrast, Formo exhibited a significant inhibitory effect on both osimertinib sensitive and resistant cells (Fig. 1d). Moreover, the MTS result showed that when concentration less than 10 μM, Formo did not decrease the cell viability of HBE and NL20 cells (Fig. 1d), suggesting that Formo is well tolerated in normal epithelial cells. We further examined colony formation of NSCLC cells with Formo or osimertinib treatment using soft agar assay. The results showed that osimertinib inhibited the anchorage-independent cell growth of osimertinib sensitive cells, but not the resistant cells as expected. Consistent with the MTS data, Formo decreased the colony formation of HCC827, H3255, H1975, A549, H1299, and HCC827 OR cells at various concentrations (Fig. 1E AND F, Supplementary Figure 1A-E). Our results suggest that Formo inhibits cell growth of both osimertinib sensitive and resistant NSCLC cells.

### Formo suppresses EGFR signaling ex vivo and in vitro

**Fig. 2:**
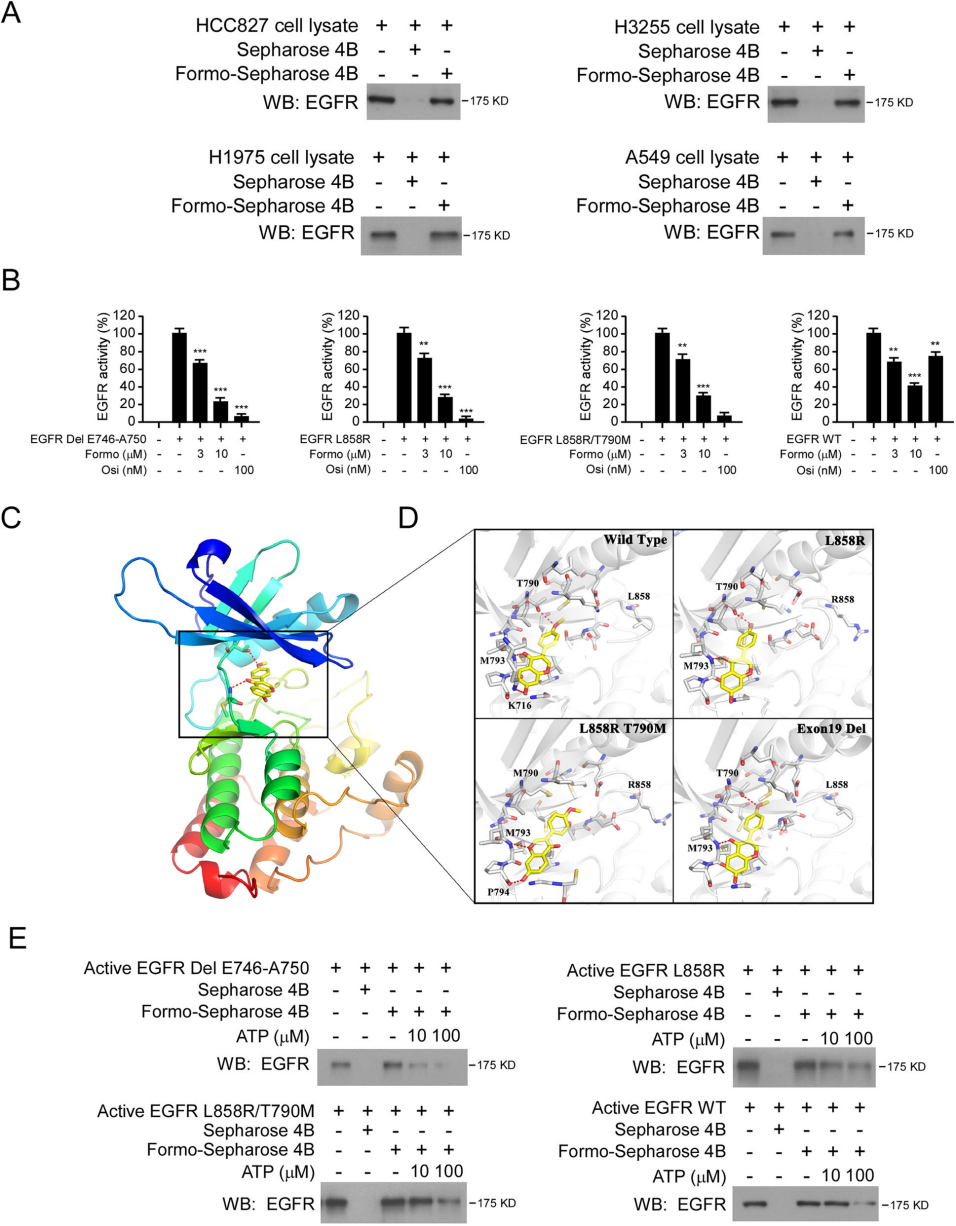
Formo inhibits the kinase activity of EGFR. **a**, Formo binds with EGFRs. Formo-Sepharose 4B beads or Sepharose 4B beads only was incubated with the whole-cell lysates (500 μg) from HCC827, H3255, H1975, or A549 cells overnight at 4 °C, and subjected to immunoblotting (IB) analysis. **b**, Formo suppresses the activity of EGFR wild type (WT) and EGFR activating mutants in vitro. Osi serves as a positive or negative control. **p < 0.01, ***p < 0.001. **c** and **d**, Binding modes of Formo with WT and mutated EGFRs predicted by molecular docking. **c**, Cartoon representation of the Formo binding pocket in EGFR. **d**, Different binding modes of Formo with 4 types of EGFR. The ligands were shown in yellow sticks, while proteins were depicted in cartoon representation with key residues indicated as gray sticks and labeled. Hydrogen bonds were shown as red dashed lines. **e**, In vitro ATP competitive binding of Formo and EGFR WT or EGFR activating mutants, were examined by pulldown and ATP competition assays

To determine the anti-tumor mechanisms of Formo, we first examined the effect of Formo on EGFR signaling. Using the in vitro pulldown assay, we analyzed the interaction between Formo and EGFR WT/activating mutants (Del E746-A750, L858R, L858R/T790M) ex vivo. By incubation with the whole-cell lysates from HCC827, H1975, H3255, and A549 (Fig. 2a), we demonstrated that EGFRs were pulled down by Formo-conjugated Sepharose 4B beads but not by Sepharose 4B beads alone, indicating that both WT EGFR and mutant EGFRs can bind with Formo. The in vitro kinase assay showed that both Formo and osimertinib significantly decreased the kinase activity of activating mutants, Del E746-A750, L858R, and L858R/T790M. Furthermore, Formo, but not osimertinib, impaired the kinase activity of EGFR WT (Fig. 2b).

We next performed molecular modeling to analyze the potential different binding modes between Formo and EGFRs. Formo was docked into the ATP-binding pocket of various forms of EGFR, including the wild type, L858R single mutation, L858R/T790M double mutations, and exon 19 deletion. Then the binding poses were refined and calculated binding free energy (ΔG). The predicted trend of ΔG for these types of EGFR was in accordance with the experimental results. Their values were − 43.6, − 48.8, − 45.1 and − 50.9 kcal/mol, respectively. As shown in Fig. 2c, the docking poses suggested that Formo could penetrate deeply into the pocket and form a hydrogen bond with the backbone nitrogen of Met793 in the hinge region in all 4 forms of EGFR. Moreover, the hydrogen bonding between Thr790 and the methoxyl of Formo was also critical. In the L858R/T790M mutated EGFR pocket, although Formo can form a hydrogen bond with the hinge region, it might take a different binding mode to avoid a stereo clash with Met790, which resulted in weaker binding affinity than the L858R single mutation. Additionally, both wild type and exon 19 deleted EGFRs form hydrogen bonding by Thr790 (bond lengths 3.3 Å and 2.9 Å, respectively). However, the wild type EGFR made one “unnecessary” hydrogen bond between Lys716 and the ligand, which hindered Formo from deeper insertion into the pocket and resulted in less potency (Fig. 2d). These results suggested that Formo was a good hit for inhibition of EGFR, especially for the mutated types of exon 19 deletion, L858R single mutation, and L858R/T790M mutation. We further conducted ATP competition assay with Formo-conjugated Sepharose 4B beads. The result showed that the binding between Formo and EGFR WT or mutants was decreased in the presence of ATP (Fig. 2e), indicating that interaction with Formo might disturb the interaction between EGFRs and ATP.

**Fig. 3:**
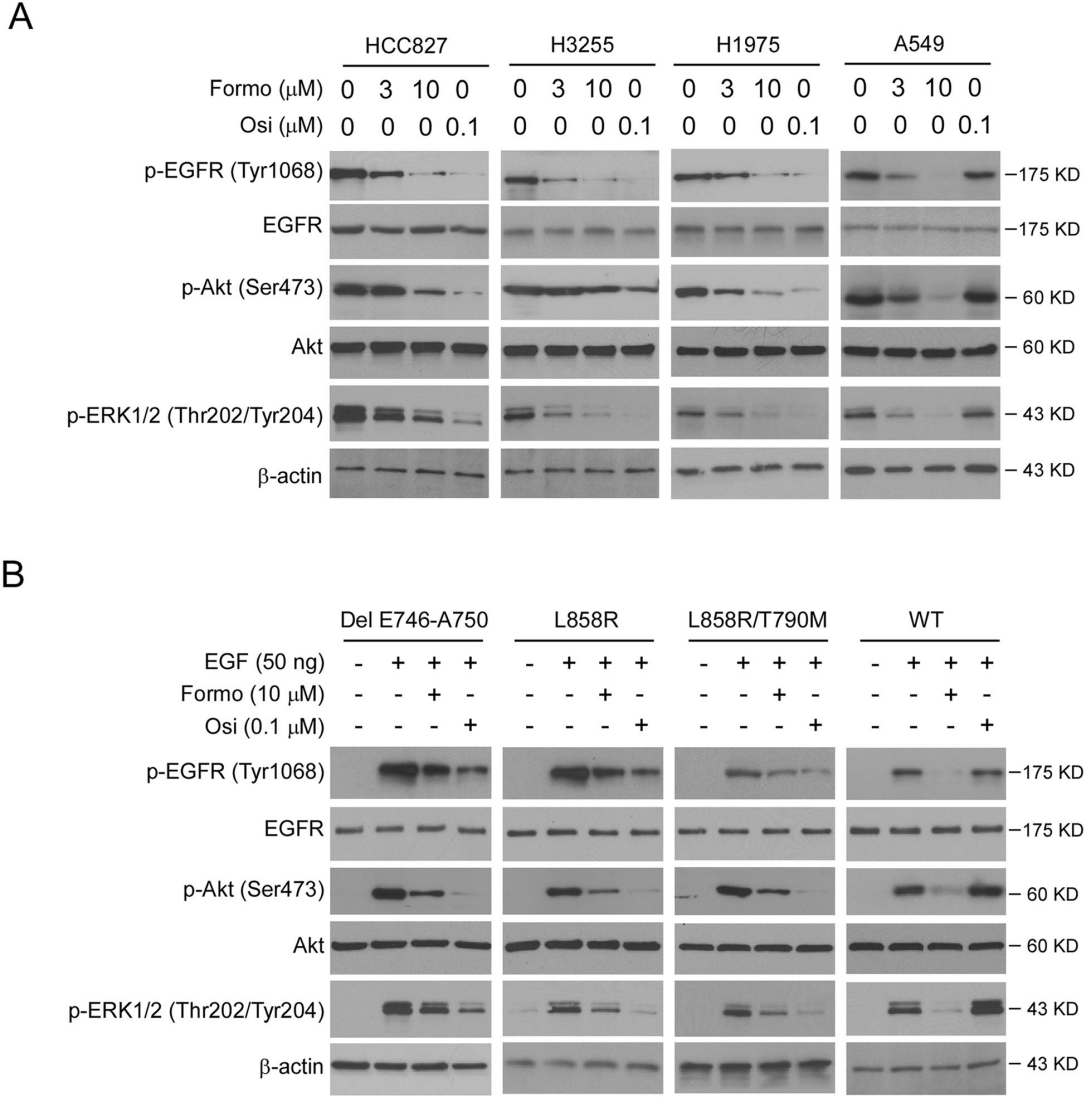
Formo inhibits EGFR signaling. **a**, Formo inhibits EGFR signaling in NSCLC cells. HCC827, H3255, H1975, and A549 cells were treated with Formo and Osi, the whole-cell lysates (WCE) were subjected to IB analysis with the antibodies as indicated. β-actin serves as a loading control. **b**, Formo inhibits EGFR signaling in Ba/F3 cells. EGFR WT or EGFR activating mutants expressing Ba/F3 cells were starved overnight with 0.1% FBS containing cell culture medium. Cells were pre-treated with Formo or Osi for 2 h, and stimulated with 50 ng EGF for 15 min, WCE was prepared and subjected to IB analysis. β-actin serves as a loading control

We next determined the effect of Formo on EGFR signaling in NSCLC cells. The IB data showed that EGFR phosphorylation was decreased in Formo treated HCC827, H3255, H1975, and A549 cells (Fig. 3a). Consistently, the phosphorylation of EGFR downstream targets, Akt (Ser473) and ERK1/2 (Thr202/Tyr204) were reduced robustly. In contrast, osimertinib only inhibited the activation of EGFR signaling in HCC827, H3255, and H1975, but not A549 cells (Fig. 3a). We further generated stable cell lines of Ba/F3 cells carrying various EGFRs, including WT, L858R, L858R/T790M, and Del E746-A750 mutants. Our data showed that Formo inhibited the phosphorylation of EGFR, Akt, and ERK1/2 in these stable cell lines (Fig. 3b). Furthermore, osimertinib exhibited more robust inhibitory efficacy on EGFR signaling in L858R, L858R/T790M, and Del E746-A750 expressing Ba/F3 cells when compare to Formo treatment. However, osimertinib failed to suppress EGFR signaling in WT EGFR expressing stable cells (Fig. 3b). These results indicate that Formo inhibits both WT and mutant EGFR signalings in NSCLC cells.

### Downregulation of Mcl-1 is required for Formo induced apoptosis in NSCLC cells

**Fig. 4:**
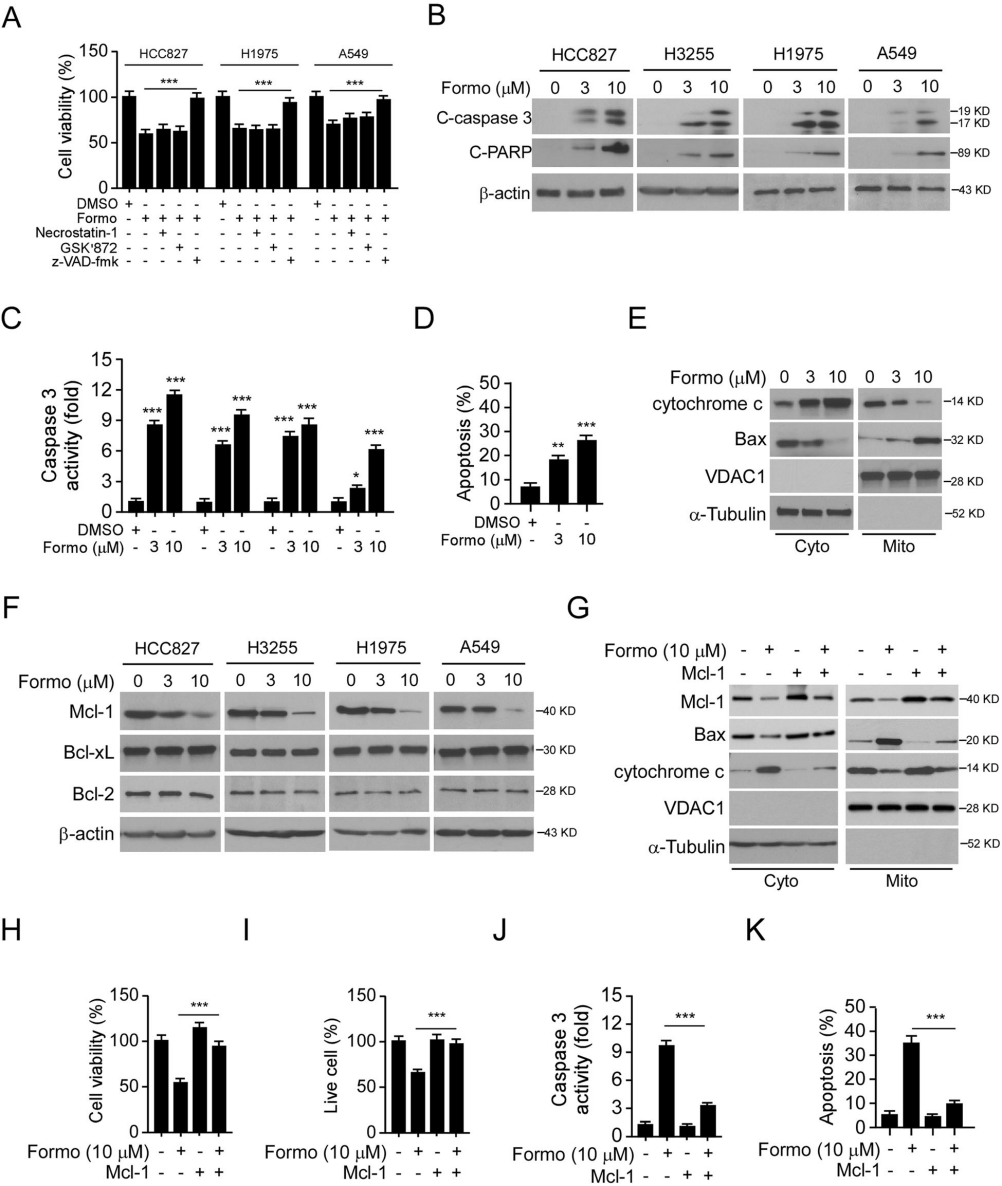
Formo reduces Mcl-1 protein level and induces apoptosis in NSCLC cells. **a**, z-VAD-fmk compromised Formo decreased cell viability. HCC827, H1975, and A549 cells were pre-treated with z-VAD-fmk, necrostatin-1, or GSK’872 for 2 h, followed by Formo (10 μM) treatment for 24 h, cell viability was analyzed by MTS assay. **b-d**, Formo promotes apoptosis in NSCLC cells. NSCLC cells were treated with Formo for 24 h, WCE was subjected to IB analysis (**b**), and caspase 3 activity analysis (**c**). Flow cytometry analysis was performed to examine the population of apoptotic cells (**d**). **e**, HCC827 cells were treated with Formo for 24 h, subcellular fractions were isolated and subjected to IB analysis. Cyto, cytoplasmic fraction; Mito, Mitochondrial fraction. **f**, NSCLC cells were treated with Formo for 24 h, WCE was subjected to IB analysis. **g**, HCC827 cells were transfected with Mcl-1 and treated with Formo for 24 h. Subcellular fractions were isolated and subjected to IB analysis. H-K, HCC827 cells were transfected with Mcl-1 and treated with Formo for 24 h, the cell viability was analyzed by MTS assay (**h**), live cell population was determined by trypan blue exclusion assay (**i**). WCE was prepared and subjected to caspase 3 activity analysis (**j**). The apoptotic cell was determined by flow cytometry (**k**). C-PARP, cleaved-PARP; C-caspase 3, cleaved- caspase 3

Activation of EGFR signaling is required for maintaining cell growth and survival in EGFR activating mutation harbored NSCLC cells. Because Formo reduced cell viability significantly, we then determined whether treatment with Formo caused cell death. Pretreated with apoptosis inhibitor z-VAD-fmk, but not necroptosis inhibitor necrostatin-1 or GSK’872, attenuated Formo decreased cell viability (Fig. 4a). The IB result showed that Formo promoted the protein level of cleaved-PARP and -caspase 3 (Fig. 4b), further confirmed the activation of apoptosis signaling. Consistently, the activity of caspase 3 was significantly enhanced in Formo-treated NSCLC cells (Fig. 4c). The flow cytometry data revealed that Formo increased the population of apoptotic HCC827 cells dose-dependently (Fig. 4d, Supplementary Figure 2A). Moreover, Formo promoted the release of cytochrome C from mitochondria to the cytoplasm, and enhanced the mitochondrial localization of Bax (Fig. 4e), indicating the activation of intrinsic apoptosis signaling.

We next investigated whether Formo-induced intrinsic apoptosis is caused by the deregulation of pro-survival Bcl-2 family members. The IB results indicated that the protein level of Mcl-1, but not the Bcl-2 or Bcl-xL, was reduced in response to Formo treatment (Fig. 4f). To determine whether the downregulation of Mcl-1 led to apoptosis in Formo-treated cells, we overexpressed Mcl-1 in HCC827 cells. Our data showed that transient transfection of Mcl-1 attenuated Formo promoted cytochrome C release and the mitochondrial localization of Bax (Fig. 4g). Furthermore, ectopic overexpression of Mcl-1 increased cell viability (Fig. 4h) and the live cell population (Fig. 4i) when compare to Formo treatment cells. Consistently, Formo-promoted caspase 3 activation (Fig. 4j) and apoptosis (Fig. 4k, Supplementary Figure 2B) were compromised by Mcl-1 transfection. These results indicate that Formo decreased the protein level of Mcl-1 and induced mitochondrial-associated apoptosis in NSCLC cells.

### Suppression of Akt is required for Formo-induced Mcl-1 downregulation

**Fig. 5:**
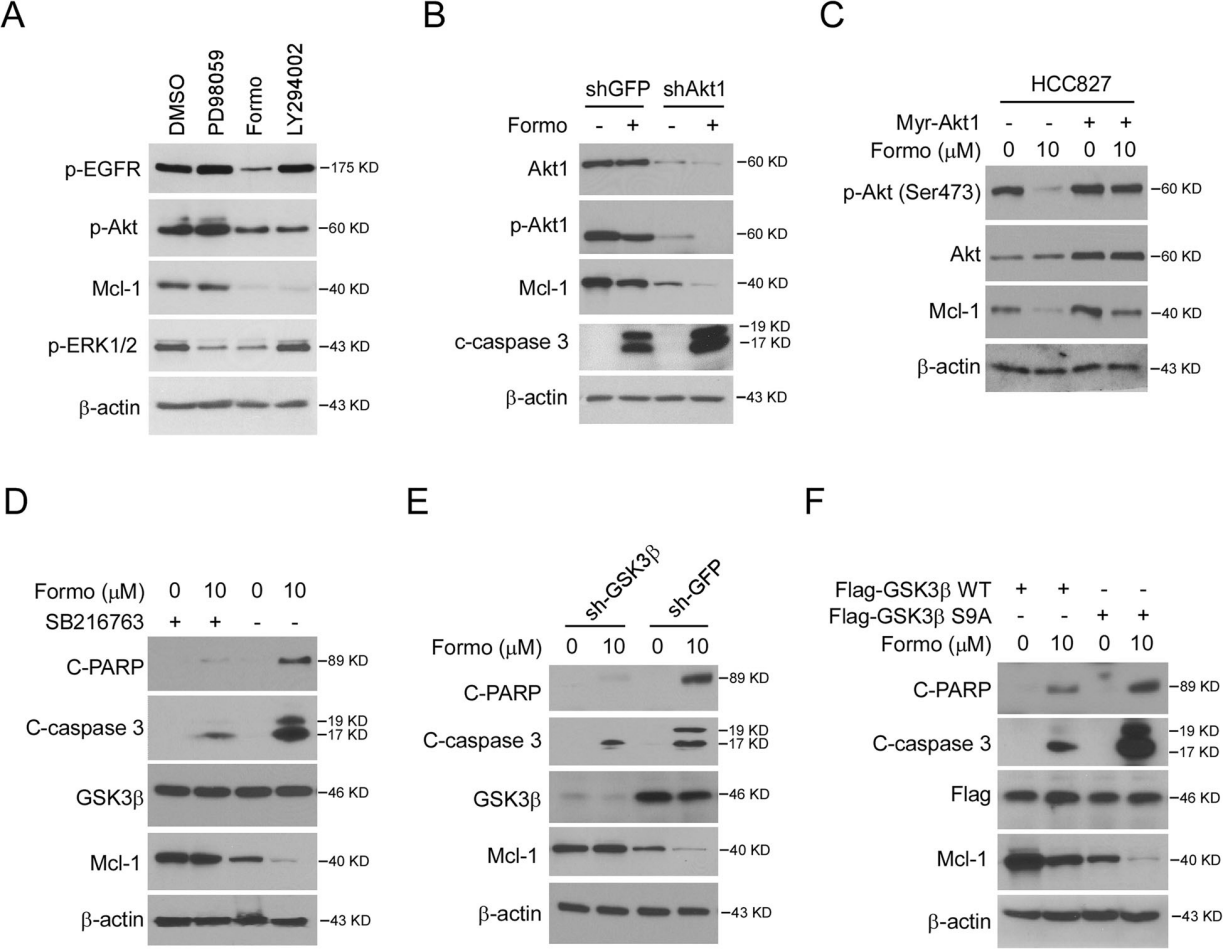
Suppression of Akt is required for Formo-induced Mcl-1 downregulation. **a**, HCC827 cells were treated with DMSO control, Formo, PD98059 (MAPK/ERK inhibitor), or LY294002 (PI3K/Akt inhibitor), WCE was subjected to IB analysis. **b**, HCC827 cells expressing shGFP or shAkt1 were treated with 10 μM Formo for 24 h, WCE was subjected to IB analysis. **c**, HCC827 cells were transfected with Myr-Akt1 and treated with 10 μM Formo for 24 h, WCE was subjected to IB analysis. **d**, HCC827 cells were pre-treated with SB216763 (GSK3 inhibitor) or DMSO control for 2 h, followed by Formo treatment for 24 h, WCE was subjected to IB analysis. **e**, HCC827 cells expressing shGFP or shGSK3β were treated with 10 μM Formo for 24 h, WCE was subjected to IB analysis. **f**, GSK3β knockout HCC827 cells were transfected with Flag- GSK3β WT or Flag-GSK3β S9A and treated with Formo for 24 h, WCE was subjected to IB analysis

ERK1/2 and Akt signalings are two major downstream oncogenic pathways of EGFR kinase. Because Formo inhibited EGFR activity and reduced Mcl-1 protein level, we therefore determined which signaling is required for Mcl-1 expression in NSCLC cells. By treating with the MAPK/ERK1/2 (PD98059) and PI3K/Akt (LY294002) pathway inhibitors, we found that suppression of Akt, but not ERK1/2, decreased Mcl-1 robustly (Fig. 5a). The silencing of Akt1 by siRNA reduced the Mcl-1 protein level and enhanced Formo-mediated Mcl-1 downregulation and cleaved-caspase 3 expression (Fig. 5b). Furthermore, the population of live cells was decreased in Formo-treated Akt knockdown cells (Supplementary Figure 3A), and the Caspase 3 activity was enhanced consistently (Supplementary Figure 3B). Moreover, ectopic overexpression of constitutively activated Akt1 (Myr-Akt) compromised Formo-induced Mcl-1 reduction (Fig. 5c). Interestingly, suppression of Akt downstream kinase GSK3β by small molecule inhibitor SB216763, restored Mcl-1 expression and decreased the protein of cleaved-caspase 3 and cleaved-PARP after Formo treatment (Fig. 5d), indicating that the reduction of Mcl-1 is dependent on Formo-activated GSK3β kinase. Likewise, depletion of GSK3β by shRNA increased Mcl-1 protein level and compromised Formo-induced apoptosis (Fig. 5e). Phosphorylation of Ser9 is required for Akt-mediated GSK3β inhibition, and loss of Ser9 phosphorylation activates GSK3β kinase [28]. We next generated the Ser9 to Ala9 (S9A) mutant and found that ectopic overexpression of GSK-3β S9A promoted Formo-induced Mcl-1 reduction and enhanced the expression of cleaved-PARP and -caspase 3 (Fig. 5f). These results suggest that downregulation of Akt and activation of GSK-3β is required for Formo-induced Mcl-1 reduction and apoptosis induction.

### Formo promotes FBW7-mediated Mcl-1 ubiquitination

**Fig. 6:**
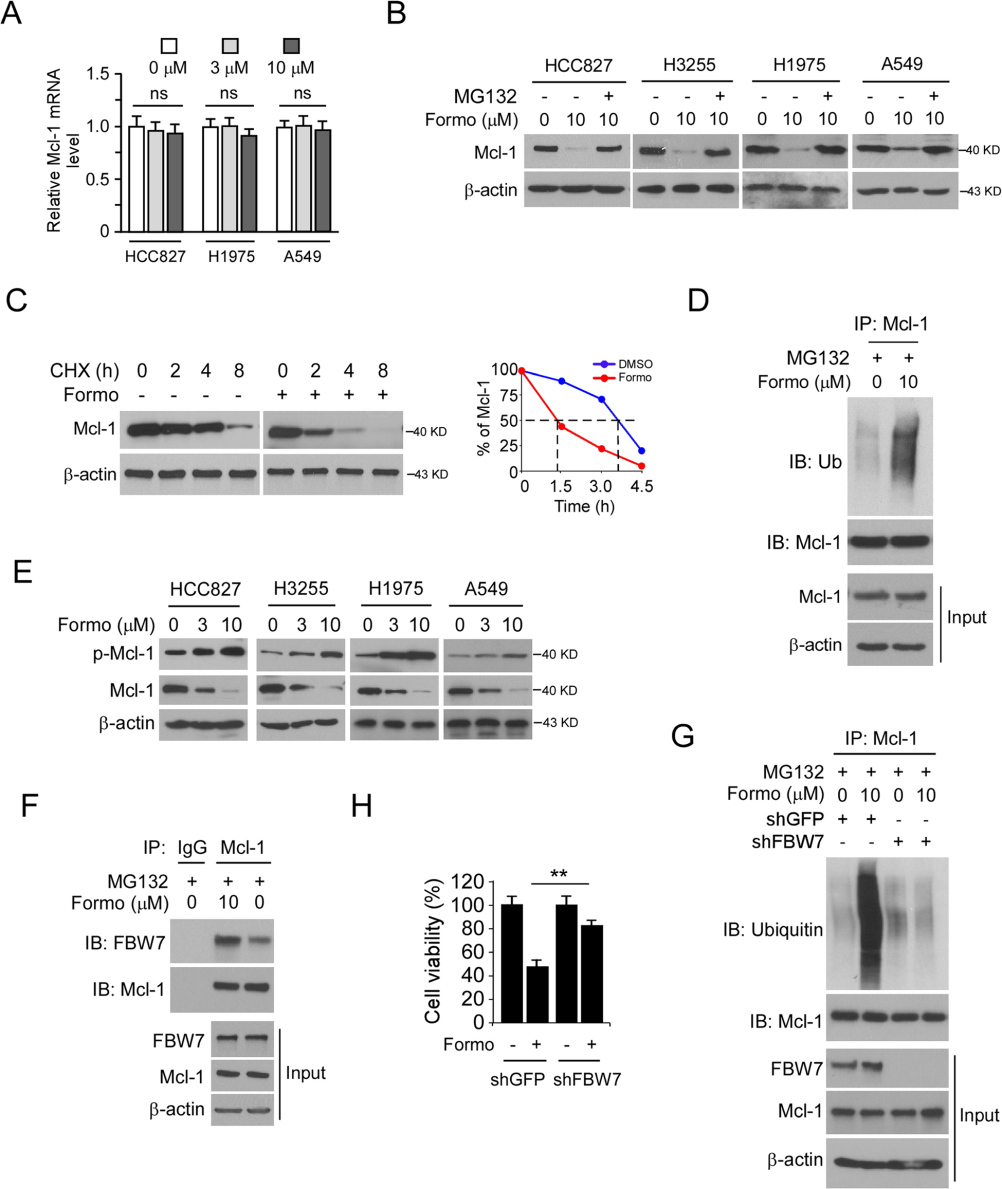
Formo promotes Mcl-1 ubiquitination in NSCLC cells. **a**, NSCLC cells were treated with Formo for 24 h, the Mcl-1 RNA level was determined by qRT-PCR. **b**, NSCLC cells were treated with Formo for 24 h, and incubated with MG132 for another 6 h. WCE was subjected to IB analysis. **c**, HCC827 cells were treated with/without Formo for 24 h, cycloheximide (CHX) was added into cell culture medium and maintained for various time points, WCE was subjected to IB analysis. **d**, HCC827 cells were treated with Formo for 24 h, followed by MG132 incubation for another 6 h. WCE was subjected to Mcl-1 ubiquitination analysis. **e**, Formo promotes Mcl-1 phosphorylation. NSCLC cells were treated with Formo for 24 h, WCE was subjected to IB analysis. **f**, HCC827 cells were treated with Formo for 24 h, followed by MG132 incubation for another 6 h. WCE was subjected to co-IP assay and IB analysis. G and H, HCC827 cells expressing shGFP or shFBW7 were treated with 10 μM Formo for 24 h, followed by MG132 incubation for another 6 h, WCE was subjected to Mcl-1 ubiquitination analysis (**g**). The cell viability was examined by MTS assay (**h**)

To better understand how GSK-3β downregulates Mcl-1 protein level, we first examined the effect of Formo on Mcl-1 transcription. Our data indicated that Formo did not inhibit Mcl-1 mRNA level significantly (Fig. 6a). However, treated with proteasome inhibitor MG132 restored Mcl-1 expression even in the presence of Formo (Fig. 6b). Moreover, treatment with Formo shortens Mcl-1 half-life from around 3.5 h to 1.5 h (Fig. 6c). These results suggest that Formo cause Mcl-1 ubiquitination and degradation. Indeed, the IB analysis revealed that treatment with Formo increased Mcl-1 endogenous polyubiquitination robustly (Fig. 6d). Phosphorylation of Mcl-1 on S159 reduced Mcl-1 stability. Our data revealed that Mcl-1 S159 phosphorylation was increased upon Formo treatment in NSCLC cells (Fig. 6e). The E3 ligase SCF^Fbw7^ promotes Mcl-1 ubiquitination in a GSK3β-mediated Mcl-1 phosphorylation-dependent manner. We speculated that FBW7 is required for Formo enhanced Mcl-1 ubiquitination. Indeed, treatment with GSK3β inhibitor SB216763 reduced Mcl-1 S159 phosphorylation and increased Mcl-1 protein level (Supplementary Figure 4). Furthermore, the interaction between Mcl-1 and FBW7 was enhanced by Formo treatment (Fig. 6f). To further confirm that FBW7 plays a crucial role in Formo-induced Mcl-1 degradation, we constructed FBW7 knockdown stable cell line. The result revealed that Formo promoted Mcl-1 ubiquitination in FBW7 proficient HCC827 cells, but failed to do so in FBW7 deficient cells (Fig. 6g). Consistently, depletion of FBW7 by shRNA increased cell viability even in the presence of Formo (Fig. 6h). These data support the notion that FBW7 is required for Formo-induced Mcl-1 ubiquitination and degradation in NSCLC cells.

### Formo inhibits xenograft tumor growth in vivo

**Fig. 7:**
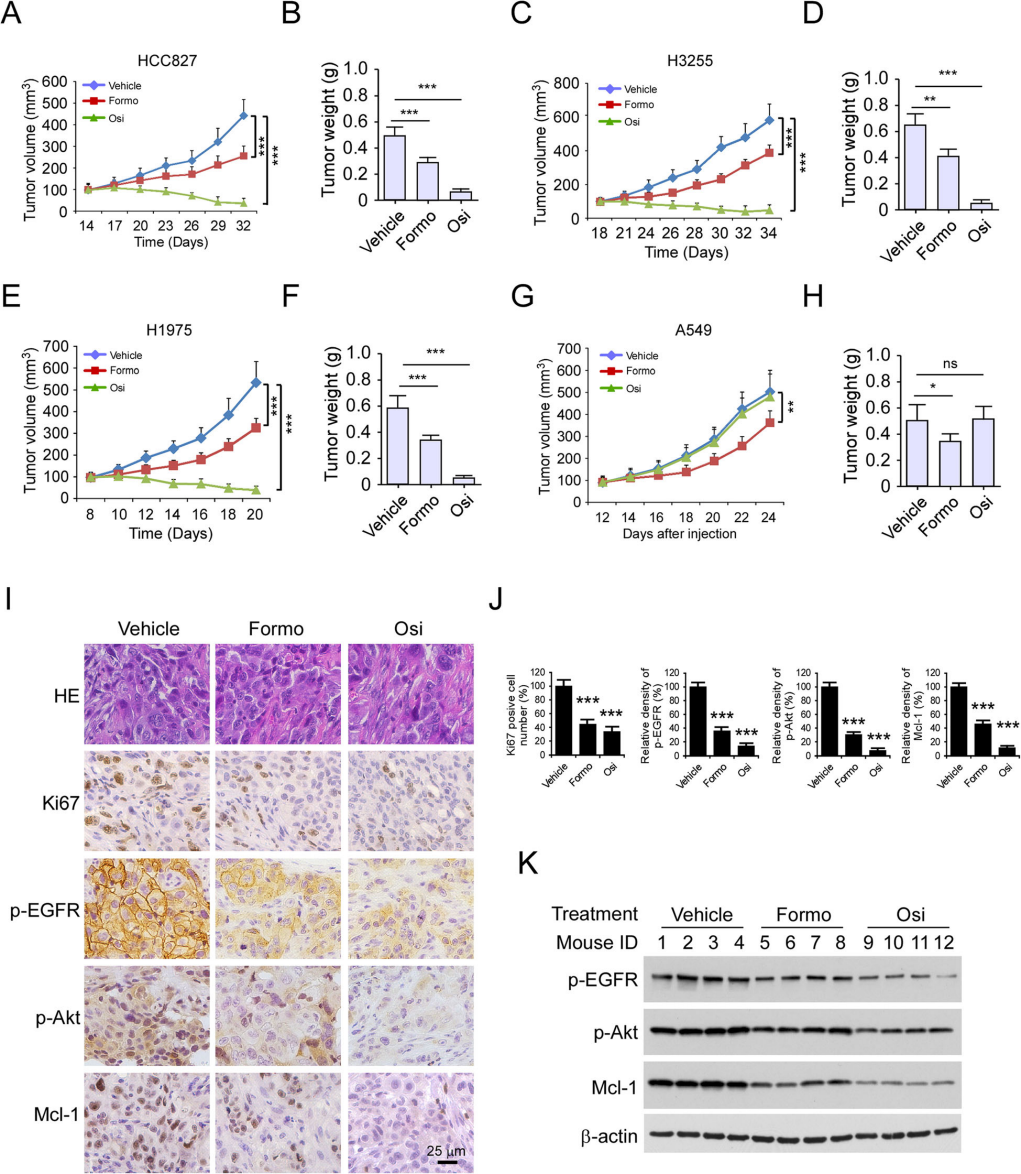
Formo inhibits in vivo tumor growth. **a** and **b**, Formo suppresses the in vivo tumor development of HCC827 cells. The tumor volume (**a**) and tumor weight (**b**) of HCC827-derived xenograft tumors treated with vehicle control, Formo, or Osi. C and D, Formo suppresses the in vivo tumor development of H3255 cells. The tumor volume (**c**) and tumor weight (**d**) of H3255-derived xenograft tumors treated with vehicle control, Formo, or Osi. E and F, Formo suppresses the in vivo tumor development of H1975 cells. The tumor volume (**e**) and tumor weight (**f**) of H1975-derived xenograft tumors treated with vehicle control, Formo, or Osi. G and H, Formo suppresses the in vivo tumor development of A549 cells. The tumor volume (**g**) and tumor weight (**h**) of A549-derived xenograft tumors treated with vehicle control, Formo, or Osi. **i**, Immunohistochemistry staining analysis of Ki67, p-EGFR, p-Akt, and Mcl-1 in HCC827-derived xenograft tumors. **j**, The qualification analysis of IHC staining of Ki67, p-EGFR, p-Akt, and Mcl-1. **k**, IB analysis of the expression of p-EGFR, p-Akt, and Mcl-1 in HCC827-derived xenograft tumors with vehicle control, Formo, or Osi treatment. *p < 0.05, **p < 0.01, ***p < 0.001. ns, not statistically significant. Scale bar, 25 μm

We next examined the in vivo anti-tumor effect of Formo using the xenograft mouse model. The results revealed that once-daily dosing of osimertinib only delayed in vivo tumor development in the HCC827, H3255, and H1975 xenografts (Fig. 7a-f, Supplementary Figure 5A-C) which harbored EGFR activating mutation. In contrast, Formo suppressed both EGFR mutant and WT tumors, including HCC827, H3255, H1975, A549, and H1299-derived xenograft (Fig. 7a-h, Supplementary Figure 5A-G). Osimertinib treated A549 and H1299-derived xenograft tumors exhibited similar tumor volumes and tumor weight as the vehicle-treated group. In contrast, Formo reduced tumor sizes significantly at the treatment endpoint than that of the vehicle treatment (Fig. 7g-h, Supplementary Figure 5D-G). However, osimertinib possessed a more potent anti-tumor efficacy than Formo in EGFR activating mutation xenografts, including HCC827, H3255, and H1975 tumors, and the tumor volume was less than 100 mm^3^ at the endpoint. We further determined the inhibitory effect of Formo on osimertinib acquired resistant HCC827 OR cells in vivo. As shown in Supplementary Figure 6H- J, Formo, but not osimertinib, reduced tumor volume and tumor weight significantly. We further performed IHC staining to examine the expression of Ki67, p-EGFR, p-Akt, and Mcl-1 in HCC827-derived xenograft tumors. The results showed that both Formo and osimertinib decreased the population of Ki67 positive cells (Fig. 7i and j). Moreover, the staining of p-EGFR, p-Akt, and Mcl-1 was reduced significantly in the Formo or osimertinib treated group (Fig. 7i and j). The IB data revealed that treatment with either Formo or osimertinib decreased the protein level of p-EGFR, p-Akt, and Mcl-1 in xenograft tumor tissues (Fig. 7k). These results consistent with our in vitro findings and further confirmed the anti-tumor effect of Formo in vivo.

To further evaluate the in vivo toxicity of Formo, we monitored mouse body weight with Formo treatment. Our data revealed that both Formo and osimertinib did not cause a significant weight loss at the current dose (Supplementary Figure 6A). The blood analysis results showed that the RBC and WBC count, as well as the AST, ALT, BUN, and Hb, were similar in Formo, osimertinib, and the vehicle-treated mice (Supplementary Figure 6B). These data indicate that Formo was well-tolerated and no significant toxicity to vital organs in vivo.

## Discussion

EGFR activating mutations serve as driver mutations and play a crucial role in the tumorigenesis of NSCLC. Therapeutic strategies using TKI to inhibit EGFR signaling have become an effective approach for these patients [1, 29]. However, most patients relapsed on TKIs due to acquired resistance. It is generally believed that hyperactivation of Akt and ERK1/2 signaling are the most common mechanisms of resistance to multiple targeted therapeutics [30]. Thus, identification of novel small molecule inhibitors to overcome primary or acquired TKI resistance is still an urgent need in NSCLC treatment. In this study, with natural compound screening, we identified Formo acted as a potential EGFR inhibitor to suppress NSCLC cells. Our data indicated that Formo docked into the EGFR ATP-binding pocket and decreased EGFR kinase activity. The in vitro and in vivo data further confirmed this inhibitory effect. Moreover, Formo reduced Mcl-1 protein level in an Akt kinase-dependent manner and promoted mitochondrial apoptosis. Our data extend the understanding of the Formo-mediated anti-tumor mechanism and suggest that suppression of both EGFR signaling and downstream oncoprotein is an alternative strategy to enhance the efficacy of the anti-tumor agent.

As the unique pro-survival Bcl-2 family member, Mcl-1 is required for maintaining cell growth and survival of NSCLC, prostate, colorectal, liver, and gastric cancer cells [10, 31]. Mcl-1 is a short half-life protein, and the expression of Mcl-1 is regulated at multiple levels, including transcription, translation, and post-translation [32]. Mcl-1 inhibits mitochondrial outer membrane permeabilization (MOMP) and suppresses cytochrome c release from mitochondria to antagonist apoptosis. Thus, overexpression of Mcl-1 was frequently observed in therapeutic resistant tumors. NF-κB dependent upregulation of Mcl-1 confers chemo/radiotherapy resistance in NSCLC and esophageal carcinoma [33]. The reduction of Mcl-1 protein by GSK3β- β-TrCP axis-mediated Mcl-1 ubiquitination and degradation enhances tumor-killing efficacy of chemotherapy agents [34]. Furthermore, degradation of Mcl-1 in colon cancer is required for targeted therapeutics induced tumor suppression [35]. Recent studies revealed that the decrease of Mcl-1 plays a crucial role in TKI or other targets therapy-mediated tumor suppression. For example, TKI promotes Mcl-1 degradation and, in combination with Bcl-XL/Bcl2 inhibitors, induced apoptosis in cancer cells [36]. Inhibition of EGFR by TKI, erlotinib, disrupts the interaction between Bim and Mcl-1 and sensitizes tumor cells to ABT-737 treatment [37]. Degradation of Mcl-1 overcome acquired resistance to osimertinib in EGFR-mutant lung cancer [38, 39]. These reports indicate that target Mcl-1 is a promising anti-tumor strategy for clinical treatment. However, there is no Mcl-1 inhibitors have been approved currently, especially the small compound, which can directly reduce the Mcl-1 protein level by ubiquitination-mediated degradation [40]. Our results revealed that Formo is a well tolerate compound that decreased Mcl-1 expression through ubiquitination-dependent degradation. Formo enhances the interaction between Mcl-1 and E3 ligase, therefore shortens Mcl-1 half-life and promotes protein destruction. These findings offer an alternative strategy to degrade the non-enzymatic activity oncoproteins through ubiquitination.

Currently, four different E3 ligases have been identified as Mcl-1 negative regulators to attenuate Mcl-1 stability, including β-Trcp [34], Trim17 [41], Mule [42], and FBW7 [43]. In contrast, multiple deubiquitinase, such as DUB3 [44], USP9X [45], and USP13 [46], were required for reversing Mcl-1 ubiquitination. In this study, we found that Formo-induced Mcl-1 degradation in an FBW7-dependent manner, depletion of FBW7 compromised Formo-promoted Mcl-1 ubiquitination. Importantly, we demonstrated that Formo suppresses EGFR-Akt signaling, which in turn activates GSK3β and eventually enhanced GSK3β-mediated Mcl-1 phosphorylation and FBW7-mediated degradation. Indeed, depletion of FBW7 causes the upregulation of Mcl-1 and confers TKI resistance in PC9 cells [47]. Our data are consistent with the previous reports and indicate that the decrease of Mcl-1 is a promising strategy to overcome TKI resistance in NSCLC treatment.

Formo is a flavonoid which originates mainly from red clovers and the Chinese herb *Astragalus membranaceus*. Accumulating evidence suggests that Formo was a multiple kinase inhibitor. A panel of kinases was identified as the protein targets of Formo, including MAPK (ERK1/2, P38), PI3K/Akt, VEGFR, FGFR, and mTOR [14, 15]. Our data showed that Formo suppressed EGFR signaling and decreased the protein level of Mcl-1. However, we cannot exclude the possibility that other oncogenic signalings are involved in Formo-mediated anti-tumor activity. Currently, numerous preclinical investigations have demonstrated that Formo exhibits chemopreventive and therapeutic potentials in multiple cancer models. The combination with Formo, significantly enhanced the anti-tumor effect of chemotherapeutic agents in a wide range of human cancers [14, 15]. Our data showed that Formo reduced the in vivo tumor growth in both EGFR WT and mutant xenograft tumors. Importantly, Formo is well-tolerated in vivo and exhibited no significant toxicity to vital organs, indicating that Formo is a potential anti-tumor candidate compound for EGFR WT and activating mutation NSCLC treatment.

## Conclusion

Overall, this study demonstrated that Formo acts as an inhibitor for the EGFR-Akt axis, and promotes FBW7-mediated Mcl-1 ubiquitination is required for the anti-tumor activity of Formo both in vitro and in vivo. Our findings suggest that targeting protein degradation might a new option for cancer treatment.

## Supplementary information

**Additional file 1: Figure S1**. The effect of formononetin (Formo) on anchorage-independent cell growth of NSCLC cells. H3255 (A), H1975 (B), A549 (C), H1299 (D), and HCC827 OR (E) cells were treated with Formo or osimertinib (Osi), anchorage-independent cell growth was analyzed by soft agar assay. Scale bar, 200 μm. Osi, osimertinib. **Figure S2**. Flow cytometry analysis of apoptotic cell population in Formo-treated HCC827 cells. A, HCC827 cells were treated with Formo for 24 h, apoptotic cells were analyzed by flow cytometry. B, HCC827 cells were transfected with Mcl-1 and treated with Formo (10 μM) for 24 h, apoptotic cells were analyzed by flow cytometry. **Figure S3**. Akt inhibition or depletion promotes Formo-induced apoptosis. A, HCC827 cells expressing shGFP or shAkt1 were treated with 10 μM Formo for 24 h, the live cell population was determined by trypan blue exclusion assay. B, HCC827 cells expressing shGFP or shAkt1 were treated with 10 μM Formo for 24 h, whole cell extract was prepared and subjected to caspase 3 activity analysis. ***, *p* < 0.001. **Figure S4**. GSK3β regulates Mcl-1 phosphorylation and expression. HCC827 cells were treated with SB216763 for 24 h, whole cell extract was subjected to immunoblotting (IB) analysis. **Figure S5**. Formo inhibits the in vivo tumor growth of NSCLC cells. A-D, The image of HCC827- (A), H3255- (B), H1975- (C), and A549 (D)-derived xenograft tumors with the vehicle control, Formo, or Osi treatment. E-G, Formo inhibits the in vivo tumor growth of H1299 cells. Tumor volume (E), the image of tumor mass (F), and tumor weight (G) of H1299 xenograft tumors treated with the vehicle control, Formo, or Osi. H-J, Formo inhibits the in vivo tumor growth of HCC827 OR cells. Tumor volume (H), the image of tumor mass (I), and tumor weight (J) of HCC827 OR xenograft tumors treated with the vehicle control, Formo, or Osi. **, *p* < 0.01; ***, *p* < 0.001. ns, not statistically significant. Scale bar, 1 cm. **Figure S6**. Toxicity analysis for treatment with Formo and Osi. A, Body weight of HCC827 xenograft tumor bearing mice with the vehicle, Formo, or Osi treatment. B, Blood analysis of mice with the vehicle, Formo, or Osi treatment. ns, not statistically significant. **Table S1**. Screened compound list.

## Data Availability

Materials are available upon request.

## Abbreviation

EGFR: Epidermal growth factor receptor
NSCLC: Non-small cell lung cancer
Mcl-1: Myeloid cell leukemia sequence 1
Formo: Formononetin
TKI: Tyrosine kinase inhibitors
Osi: Osimertinib
PFS: Progression-free survival
WT: Wild type
PDB: Protein Data Bank
DOPE: Discrete Optimized Protein Energy
ALK: Anaplastic lymphoma kinase
MOMP: Mitochondrial outer membrane permeabilization
IB: Immunoblotting
IHC: Immunohistochemical staining
CHX: Cycloheximide
Cyto: Cytoplasmic fraction
Mito: Mitochondrial fraction
RBC: Red blood cells
WBC: White blood cells
Hb: Hemoglobin
ALT: Alanine aminotransferase
AST: Aspartate aminotransferase
BUN: Blood urea nitrogen
