# Supplementary material for: Formononetin inhibits tumor growth by suppression of EGFR-Akt-Mcl-1 axis in non-small cell lung cancer
Source: J Exp Clin Cancer Res. 2020 Apr 10;39:62. doi: 10.1186/s13046-020-01566-2 (PMC7146989; doi:10.1186/s13046-020-01566-2)
Supplement: Supplementary file 1 — Additional file 1: Figure S1. The effect of formononetin (Formo) on anchorage-independent cell growth of NSCLC cells. H3255 (A), H1975 (B), A549 (C), H1299 (D), and HCC827 OR (E) cells were treated with Formo or osimertinib (Osi), anchorage-independent cell growth was analyzed by soft agar assay. Scale bar, 200 μm. Osi, osimertinib. Figure S2. Flow cytometry analysis of apoptotic cell population in Formo-treated HCC827 cells. A, HCC827 cells were treated with Formo for 24 h, apoptotic cells were analyzed by flow cytometry. B, HCC827 cells were transfected with Mcl-1 and treated with Formo (10 μM) for 24 h, apoptotic cells were analyzed by flow cytometry. Figure S3. Akt inhibition or depletion promotes Formo-induced apoptosis. A, HCC827 cells expressing shGFP or shAkt1 were treated with 10 μM Formo for 24 h, the live cell population was determined by trypan blue exclusion assay. B, HCC827 cells expressing shGFP or shAkt1 were treated with 10 μM Formo for 24 h, whole cell extract was prepared and subjected to caspase 3 activity analysis. ***, p < 0.001. Figure S4. GSK3β regulates Mcl-1 phosphorylation and expression. HCC827 cells were treated with SB216763 for 24 h, whole cell extract was subjected to immunoblotting (IB) analysis. Figure S5. Formo inhibits the in vivo tumor growth of NSCLC cells. A-D, The image of HCC827- (A), H3255- (B), H1975- (C), and A549 (D)-derived xenograft tumors with the vehicle control, Formo, or Osi treatment. E-G, Formo inhibits the in vivo tumor growth of H1299 cells. Tumor volume (E), the image of tumor mass (F), and tumor weight (G) of H1299 xenograft tumors treated with the vehicle control, Formo, or Osi. H-J, Formo inhibits the in vivo tumor growth of HCC827 OR cells. Tumor volume (H), the image of tumor mass (I), and tumor weight (J) of HCC827 OR xenograft tumors treated with the vehicle control, Formo, or Osi. **, p < 0.01; ***, p < 0.001. ns, not statistically significant. Scale bar, 1 cm. Figure S6. Toxicity analysis for treatment [file 13046_2020_1566_MOESM1_ESM.pdf]

Supplementary Figure legends

Figure S1. The effect of formononetin (Formo) on anchorage-independent cell growth of NSCLC cells. H3255 (A), H1975 (B), A549 (C), H1299 (D), and HCC827 OR (E) cells were treated with Formo or osimertinib (Osi), anchorage-independent cell growth was analyzed by soft agar assay. Scale bar, 200 μm. Osi, osimertinib.

Figure S2. Flow cytometry analysis of apoptotic cell population in Formo-treated HCC827 cells. A, HCC827 cells were treated with Formo for 24 h, apoptotic cells were analyzed by flow cytometry. B, HCC827 cells were transfected with Mcl-1 and treated with Formo (10 μM) for 24 h, apoptotic cells were analyzed by flow cytometry.

Figure S3. Akt inhibition or depletion promotes Formo-induced apoptosis. A, HCC827 cells expressing shGFP or shAkt1 were treated with 10 μM Formo for 24 h, the live cell population was determined by trypan blue exclusion assay. B, HCC827 cells expressing shGFP or shAkt1 were treated with 10 μM Formo for 24 h, whole cell extract was prepared and subjected to caspase 3 activity analysis. ***, $p<0.001$.

Figure S4. GSK3β regulates Mcl-1 phosphorylation and expression. HCC827 cells were treated with SB216763 for 24 h, whole cell extract was subjected to immunoblotting (IB) analysis.

Figure S5. Formo inhibits the *in vivo* tumor growth of NSCLC cells. A-D, The image of HCC827- (A), H3255- (B), H1975- (C), and A549 (D)-derived xenograft tumors with the vehicle control, Formo, or Osi treatment. E-G, Formo inhibits the *in vivo* tumor growth of H1299 cells. Tumor volume (E), the image of tumor mass (F), and tumor weight (G) of H1299 xenograft tumors treated with the vehicle control, Formo, or Osi. H-J, Formo inhibits the *in vivo* tumor growth of HCC827 OR cells. Tumor volume (H), the image of tumor mass (I), and tumor weight (J) of HCC827 OR xenograft tumors treated with the vehicle control, Formo, or Osi. **, $p<0.01$; ***, $p<0.001$. ns, not statistically significant. Scale bar, 1cm.

Figure S6. Toxicity analysis for treatment with Formo and Osi. A, Body weight of HCC827 xenograft tumor bearing mice with the vehicle, Formo, or Osi treatment. B, Blood analysis of mice with the vehicle, Formo, or Osi treatment. ns, not statistically significant.

Supplementary Figure 1

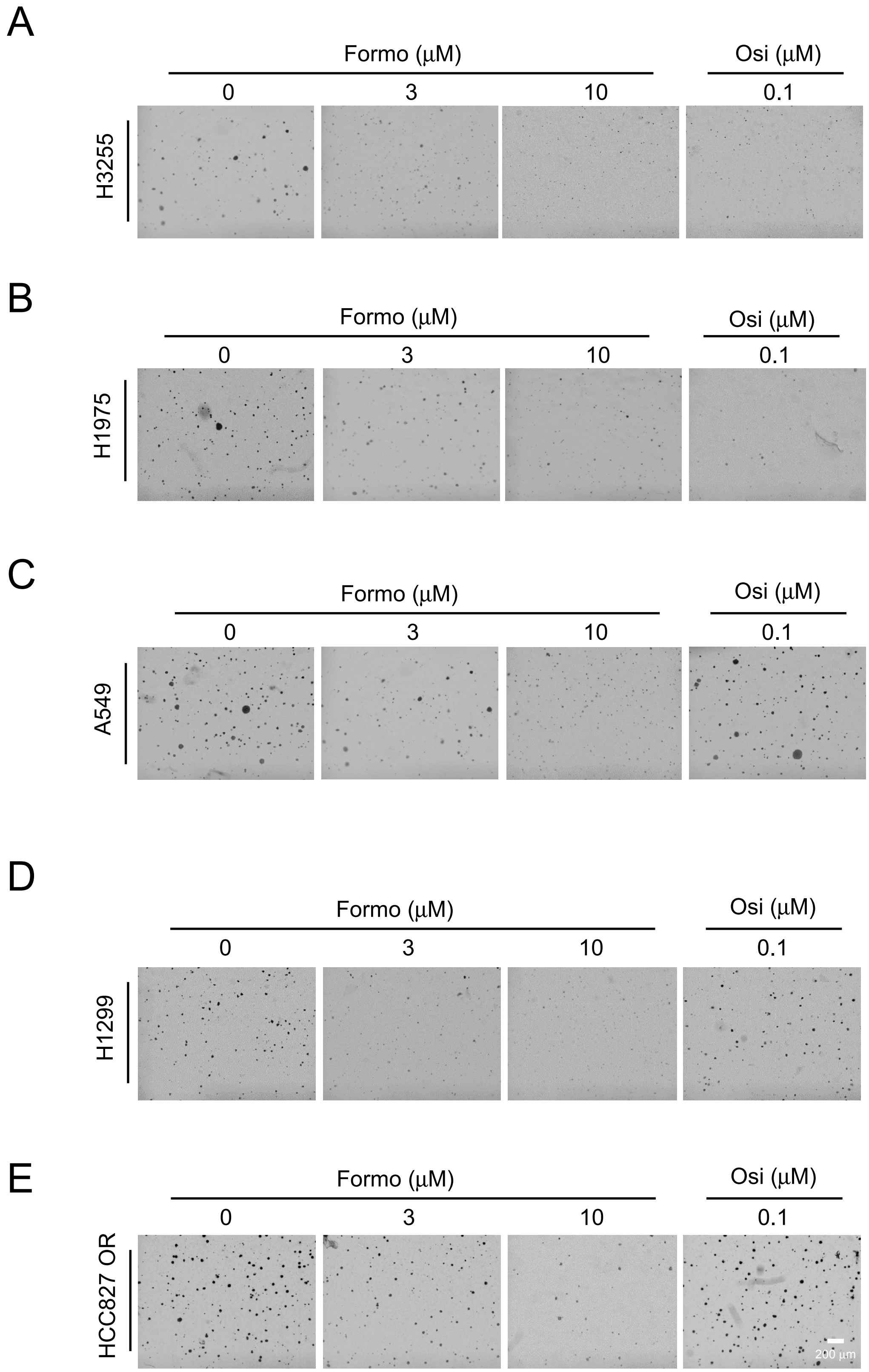

Supplementary Figure 2

A

DMSO	+	-	-
Formo (μM)	-	3	10

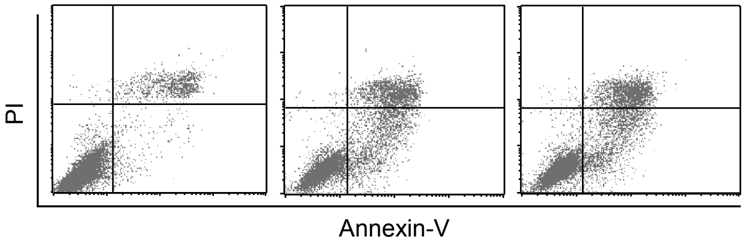

B

Formo (10 μM)	-	+	-	+
Mcl-1	-	-	+	+

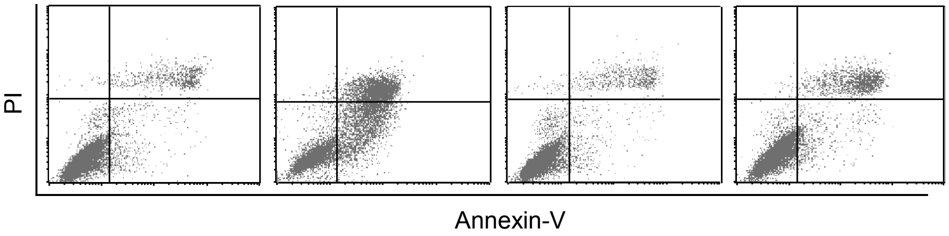

Supplementary Figure 3

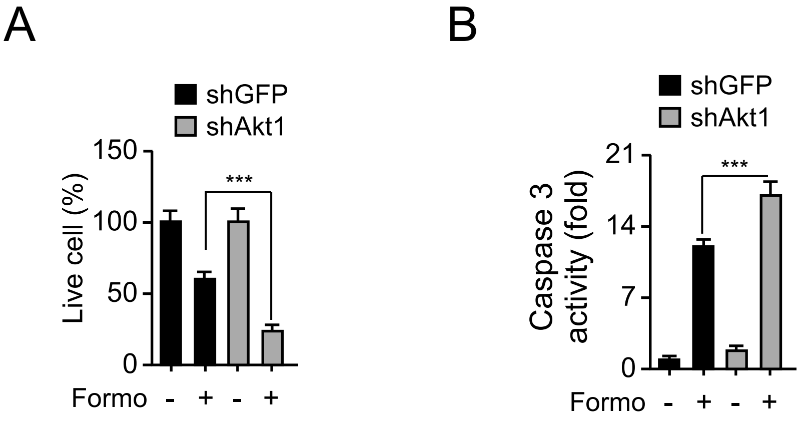

Supplementary Figure 4

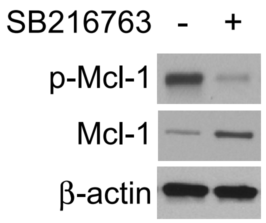

Supplementary Figure 5

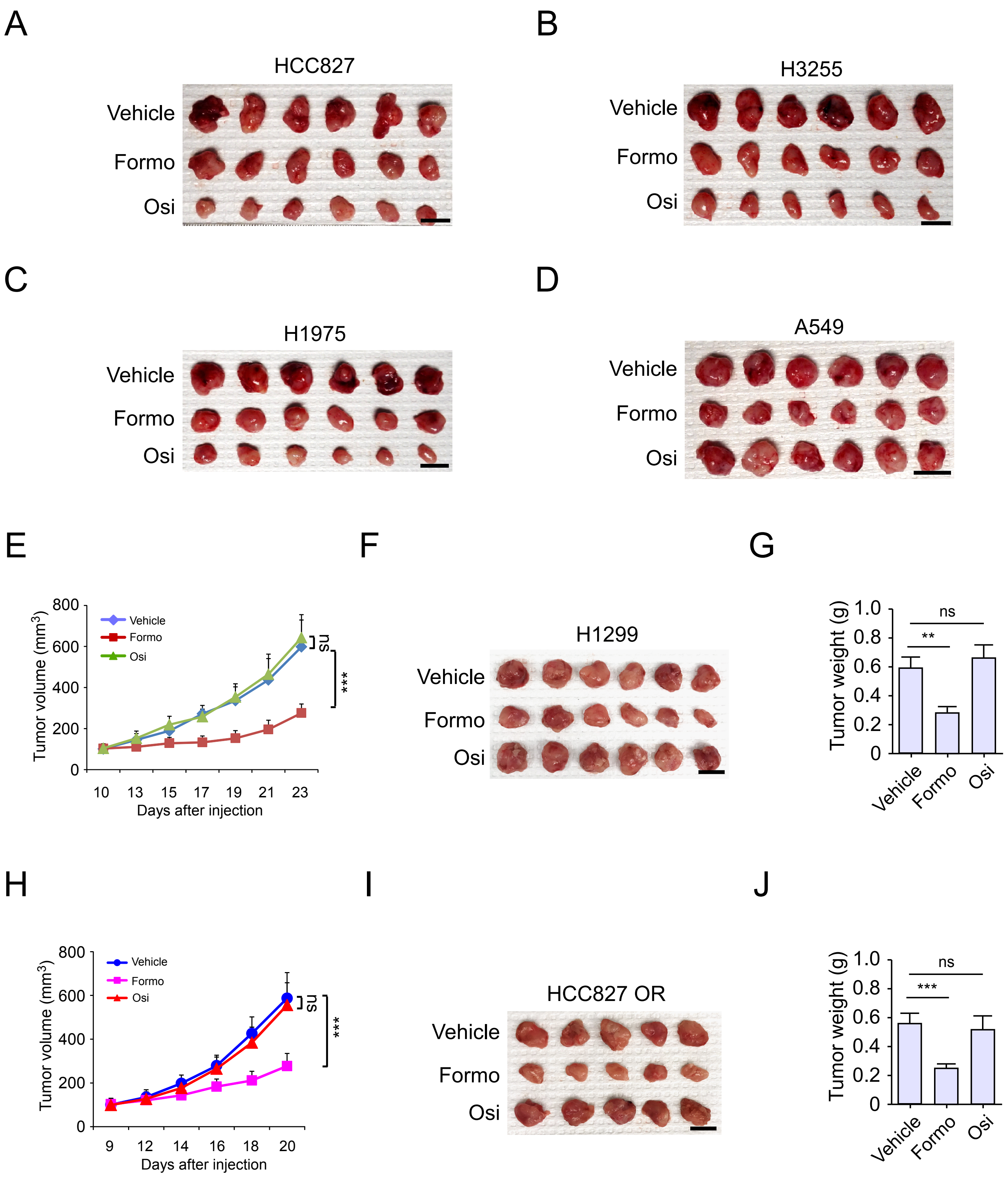

Supplementary Figure 6

A

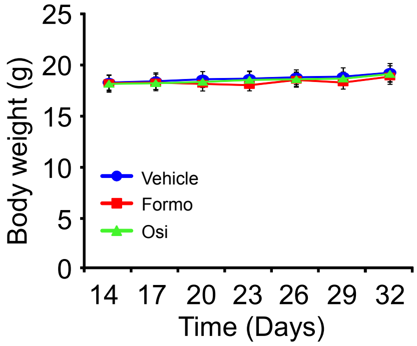

B

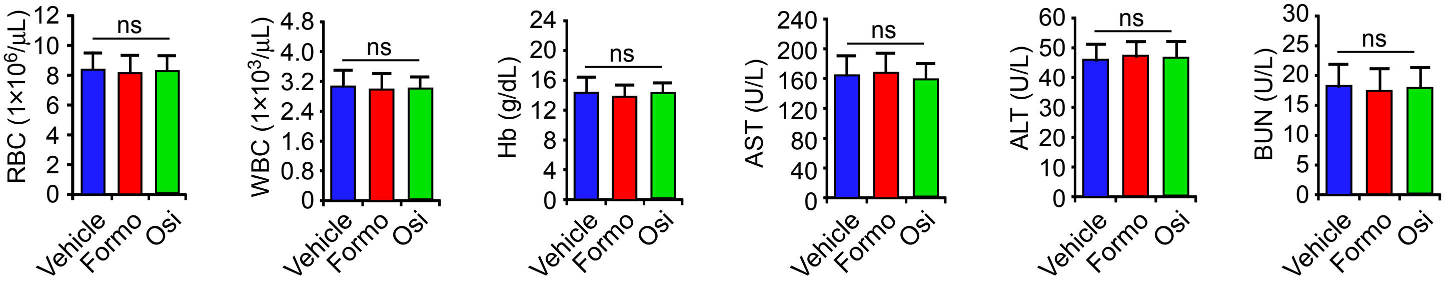

Table S1 Screened compound list

Name	Name
DMSO	Balofloxacin
Aloe-emodin	Clindamycin palmitate HCl
Alizarin	Dopamine hydrochloride (Inotropin)
Rutaecarpine (Rutecarpine)	Uridine
Puerarin (Kakonein)	Econazole nitrate (Spectazole)
Silymarin (Silybin B)	Fluconazole
Naringin (Naringoside)	Capsaicin
Baicalein	Dextrose (D-glucose)
Chrysin	Tetrahydropapaverine hydrochloride
Inosine	Artemether
Resveratrol	Oxymatrine (Matrine N-oxide)
Daphnetin	All-trans Retinoic Acid (Tretinoin)
Piperine (1-Piperoylpiperidine)	Bilobalide
4-Methylumbelliferone (4-MU)	Atropine sulfate monohydrate
Adenine hydrochloride	Voriconazole
Chlorogenic acid	Quinine hydrochloride dihydrate
Aesculin (Esculin)	Forskolin
EGCG	Xylose
Linezolid (Zyvox)	Ursodiol (Actigal Urso)
Nalidixic acid (NegGram)	Ginkgolide A
Taxifolin (Dihydroquercetin)	Azomycin (2-Nitroimidazole)
Neohesperidin dihydrochalcone (Nhdc)	Ginkgolide B
Amygdalin	Oridonin (Isodonol)
Myricetin (Cannabiscetin)	Cortisone acetate (Cortone)
Troxerutin	Sinomenine (Cucoline)
Genistein	Gatifloxacin
Osthole (Osthol)	Rotenone (Barbasco)
Novobiocin sodium (Albamycin)	Gambogic Acid
Meropenem	4'-Demethylepipodophyllotoxin
Cefoperazone (Cefobid)	L-Thyroxine
Quercetin dihydrate (Sophoretin)	Dehydroepiandrosterone (DHEA)
Tigecycline	Sclareolide (Norambreinolide)
Silibinin (Silybin)	Dexamethasone
Acarbose	Celastrol
Guanosine	5-Aminolevulinic acid hydrochloride
Albendazole (Albenza)	Artesunate
Asaraldehyde (Asaronaldehyde)	Vincristine Sulfate
Cyclocytidine HCl	Clotrimazole (Canesten)
Adenosine (Adenocard)	Roxithromycin (Roxl-150)
Mycophenolic acid	Clarithromycin (Biaxin Klacid)
Myricitrin (Myricitrine)	Shikimic acid (Shikimate)
Synephrine HCl	Idebenone
Orotic acid (6-Carboxyuracil)	Natamycin
Paeonol (Peonol)	Caffeic acid
Reserpine	2-Methoxyestradiol
Nitrofurazone (Nitrofural)	Artemisinin
Cefdinir (Omnicef)	Lincomycin hydrochloride (Lincocin)
Tebipenem pivoxil (L-084)	Formononetin
Cinchonidine	Cloxacillin sodium
L-Adrenaline (Epinephrine)	
